# Security of Information and Networks

**DOI:** 10.1155/2015/150640

**Published:** 2015-05-04

**Authors:** Iftikhar Ahmad, Aneel Rahim, Adeel Javed, Hafiz Malik

**Affiliations:** ^1^King Saud University, Saudi Arabia; ^2^Waterford Institute of Technology, Waterford, Ireland; ^3^University of Otago, Dunedin, New Zealand; ^4^University of Michigan, Dearborn, USA

This special issue aims to make people aware and up to date of the innovative research in the area of emerging techniques and methods in security of information and network. The majority of organizations in the commercial and government sectors are relying completely on their computer and network systems. Extensive attacks can cause heavy loss in a few seconds. Therefore, securing their computers, networks, and information is imperative. Based on the importance of security and interest of the researchers, this special issue is focused on attracting good work on innovative methods and techniques in order to address unique security issues which are introduced by new computing paradigms and techniques.

This special issue presents the following papers in the most active areas of research in security of information and networks. The brief introduction of the selected papers is provided.

O. Tayan et al. proposed the paper “A Hybrid Digital-Signature and Zero-Watermarking Approach for Authentication and Protection of Sensitive Electronic Documents.” This paper addresses the problems and threats associated with verification of integrity, proof-of-authenticity, tamper-detection, and copyright protection for digital-text content. The proposed algorithm was implemented and shown to be robust against undetected content modifications and is capable of confirming proof-of-originality whilst detecting and locating deliberate/nondeliberate tampering. Additionally, enhancements in resource-utilization and reduced redundancies were achieved in comparison to traditional encryption-based approaches.

Y. Liu et al., worked on the paper “Network Anomaly Detection System with Optimized DS Evidence Theory” in which a novel network anomaly detection system is proposed with Optimized Dempster-Shafer (ODS) evidence theory and Regression Basic Probability Assignment (RBPA) function. In this model, the authors add weights to each senor to optimize DS evidence theory according to its previous predicted accuracy and RBPA employs sensor's regression ability to address complex network. They proved that this network anomaly detection model has a better detection rate, and RBPA and ODS optimization methods can improve system performance significantly.

A. Soleymani et al. proposed the paper “A Chaotic Cryptosystem for Images Based on Henon and Arnold Cat Map.” In this paper, an encryption scheme is presented for securing images based on Arnold cat and Henon chaotic maps. The scheme uses Arnold cat map for bit and pixel-level permutations on plain and secret images, while Henon map creates secret images and specific parameters for the permutations. Both the encryption and decryption processes are explained, formulated, and graphically presented. The results of security analysis of five different images demonstrate the strength of the proposed cryptosystem against statistical, brute force and differential attacks. The evaluated running time for both encryption and decryption processes guarantees that the cryptosystem can work effectively in real-time applications.

S. M. Al-Saleem and H. Ullah worked on the paper “Security Considerations and Recommendations in Computer-Based Testing” in which the security considerations associated with CBT are investigated and some recommendations are given for the security of tests. A palm-based biometric authentication system is proposed and incorporated in basic authentication system (username/password) in order to check the identity and authenticity of the examinee.

Z. Moghaddasi et al. worked on the paper “Improving RLRN Image Splicing Detection with the Use of PCA and Kernel PCA.” This study focuses on improving one of the image splicing detection algorithms, that is, the Run-Length Run Number (RLRN) algorithm, by applying two dimension-reduction methods, namely, principal component analysis (PCA) and kernel PCA. Support vector machine is used to distinguish between authentic and spliced images. Results show that kernel PCA is a nonlinear dimension-reduction method that has the best effect on R, G, B, and Y channels and gray-scale images.

M. A. Saleh and A. A. Manaf proposed the paper “A Novel Protective Framework for Defeating HTTP-Based Denial of Service and Distributed Denial of Service Attacks.” This work proposes a Flexible, Collaborative, Multilayer, DDoS Prevention Framework (FCMDPF). The innovative design of the FCMDPF framework handles all aspects of HTTP-based DoS/DDoS attacks through the following three subsequent framework's schemes (layers). Firstly, an Outer Blocking (OB) scheme blocks attacking IP source if it is listed on the black list table. Secondly, the Service Trace Back Oriented Architecture (STBOA) scheme validates whether the incoming request is launched by a human or by an automated tool. Then, it traces back the true attacking IP source. Thirdly, the Flexible Advanced Entropy Based (FAEB) scheme eliminates High Rate DDoS (HR-DDoS) and Flash Crowd (FC) attacks. The proposed framework's design provides an efficient protection for web applications against all sorts of DoS/DDoS attacks.

H. Y. Lee, proposed the paper “Method for Detecting Manipulated Compilation of Sensing Reports in Wireless Sensor Networks.” In the proposed method, every sensing report is collaboratively generated and verified by cluster nodes based on very loose synchronization. Once a cluster node has detected an MCA for a real event, it can reforward a legitimate report immediately. Therefore, the event can be properly reported to the users. The performance of the proposed method is shown with analytical and experimental results.

V. Jaganathan et al. worked on the paper “Using a Prediction Model to Manage Cyber Security Threats.” As cyberattacks are an important issue faced by all organizations, securing information systems is critical. Organizations should be able to understand the ecosystem and predict attacks. Predicting attacks quantitatively should be part of risk management. The cost impact due to worms, viruses, or other malicious software is significant. This paper proposed a mathematical model to predict the impact of an attack based on significant factors that influence cyber security. This model also considers the environmental information required. It is generalized and can be customized to the needs of the individual organization.

M. M. Al-Dabbagh et al. worked on the paper “Intelligent Bar Chart Plagiarism Detection in Documents.” Plagiarism is considered as heinous electronic crime and intellectual theft. This paper introduced a new technique for extracting the features from documents which cannot be mined via Optical Character Recognition (OCR). By identifying the intimate relationship between the text and graphical components, the present technique pulls out the start, end, and exact value for each bar. Furthermore, the Word 2-gram and Euclidean distance methods are used to accurately detect and determine the plagiarism. The efficient detection of various plagiarized patterns is demonstrated. The system not only identifies copy-move forgery of bar charts but also distinguishes any possible modification applied on these images such as change of scales, colors, swapping among bars location, and even alteration on caption including summarizing and restructuring. This technique can constitute a basis for intelligent forgery detection in documents.

*Iftikhar Ahmad* *Iftikhar Ahmad* *Aneel Rahim* *Aneel Rahim* *Adeel Javed* *Adeel Javed* *Hafiz Malik* *Hafiz Malik*

